# Field evaluation of the residual efficacy of new generation insecticides for potential use in indoor residual spray programmes in South Africa

**DOI:** 10.1186/s12936-024-04963-6

**Published:** 2024-04-30

**Authors:** Rajendra Maharaj, Ishen Seocharan, Vishan Lakan, Zuziwe Nyawo, Moses Mkhabela, Yusentha Balakrishna

**Affiliations:** 1 Malaria Research Group, South African Medical Research Council, Durban, South Africa; 2 Biostatistics Research Unit, South African Medical Research Council, Durban, South Africa; 3 KwaZulu-Natal Department of Health, Malaria Control Programme, Jozini, South Africa

**Keywords:** Actellic, SumiShield, Fludora® Fusion, Indoor residual spraying, Efficacy, Insecticide resistance

## Abstract

**Background:**

The decreasing residual efficacy of insecticides is an important factor when making decisions on insecticide choice for national malaria control programmes. The major challenge to using chemicals for vector control is the selection for the development of insecticide resistance. Since insecticide resistance has been recorded for most of the existing insecticides used for indoor residual spraying, namely, DDT, pyrethroids, organophosphates and carbamates, and new chemicals are necessary for the continued success of indoor residual spraying. The aim of this study was to assess the residual efficacy of Actellic 300CS, SumiShield™ 50WG and Fludora®Fusion by spraying on different wall surfaces.

**Methods:**

One hundred and sixty-eight houses with different wall surface types (mud, cement, painted cement, and tin) which represented the rural house wall surface types in KwaZulu-Natal, South Africa were used to evaluate the residual efficacy of Actellic 300CS, SumiShield 50WG and Fludora®Fusion with DDT as the positive control. All houses were sprayed by experienced spray operators from the Malaria Control Programme. Efficacy of these insecticides were evaluated by contact bioassays against *Anopheles arabiensis*, a vector species. The residual efficacy of the insecticide formulations was evaluated against a susceptible insectary-reared population of *An. arabiensis* using WHO cone bioassays.

**Results:**

Effectiveness of the three insecticides was observed up to 12 months post-spray. When assessing the achievement of 100% mortality over time, SumiShield performed significantly better than DDT on mud (OR 2.28, 95% CI 1.72–3.04) and painted cement wall types (OR 3.52, 95% CI 2.36–5.26). On cement wall types, Actellic was found to be less effective than DDT (OR 0.55, 95% CI 0.37–0.82) while Fludora®Fusion was less effective on tin wall types (OR 0.67, 95% CI 0.47–0.95). When compared to the combined efficacy of DDT on mud surfaces, SumiShield applied to each of the mud, cement and painted cement wall types and DDT applied to the cement wall types was found to be significantly more effective. These insecticides usually resulted in 100% mortality for up to 12 months with a delayed mortality period of 96–144 h, depending on the insecticide evaluated and the surface type sprayed.

**Conclusion:**

Field evaluation of these insecticides have shown that Actellic, SumiShield and Fludora®Fusion are suitable replacements for DDT. Each of these insecticides can be used for malaria vector control, requiring just one spray round. These insecticides can be used in rotation or as mosaic spraying.

## Background

Indoor residual spraying (IRS) is used for malaria control in many countries across the African continent. Although IRS has been the mainstay of vector control in many African countries, malaria remains a significant public health challenge despite many countries in Africa aiming for elimination by 2030 [1]. Globally, malaria causes millions of cases and hundreds of thousands of deaths. In their 2023 Annual Malaria Report, the World Health Organization (WHO) reported that malaria caused 249 million infections and 608 000 deaths [1]. Africa is a major contributor to the global malaria burden, being home to 95% of malaria cases and 96% of malaria deaths [1]. This disease disproportionately affects poor, rural communities with limited health care access. Approximately, 77% of malaria deaths globally are children under 5 years of age in Africa [1].

Measures are needed to strengthen malaria control interventions that aim to eradicate malaria. IRS has been used in vector control for over 80 years in South Africa [2], whilst in many African countries, nets impregnated with pyrethroid insecticides are often used to prevent malaria transmission by forming a physical barrier between the human host and the vector mosquito [3]. IRS is one of the most effective strategies used against anopheline mosquitoes, including *Anopheles gambiae *sensu stricto (*s.s.), An. arabiensis* and *Anopheles funestus*, the main malaria vectors in Africa [4, 5]. As in many other countries, IRS has been the foundation of malaria control in southern Africa. Indoor residual spraying was first attempted in South Africa in the early 1940s and has since become the focus of malaria control activities in the country [2]. Unfortunately, due to selection pressure from insecticide use, mosquito vectors with inherent traits that enable them to survive exposure to insecticides or permits them to prevent contact with insecticides have been selected for [6].

In South Africa, the persistent indoor feeding, and resting behaviours of some mosquito species such as *An. funestus*, sustain the relevance of IRS as an effective vector control strategy. Nonetheless, the quest for malaria elimination demands new, cost-efficient, and efficacious tools. A critical need exists for an affordable alternative to dichloro-diphenyl-trichloroethane (DDT), an insecticide with prolonged effectiveness which is affordable for malaria control programmes across Africa. This requirement is underscored by the widespread development of resistance to all existing classes of insecticides, further complicating the battle against malaria [7].

Resistance to all classes of insecticides have been reported from the African continent [8, 9]. This has important implications for the continued use of these chemicals in malaria control programmes. In the southern African region, resistance to pyrethroids and DDT has been reported [10, 11]. Previously, pockets of resistance to most classes of insecticides were reported from especially KwaZulu-Natal [12] and it was recommended that insecticide resistance management as well as alternative control techniques be implemented to target outdoor-resting *An. arabiensis* in northern KwaZulu-Natal.

Since there are genes favouring insecticide resistance circulating within this population, it is only a matter of time before insecticide resistance is re-established should the vector control policy not be changed. New insecticides are required to replace the existing insecticides to ensure that mosquito populations, and hence transmission, is eliminated. Furthermore, the availability of certain chemicals used in IRS is precarious, as a few insecticides can only be sourced exclusively from single suppliers who may halt production without notice.

To address the need for new tools, a study was conducted to evaluate the field efficacy of insecticides with new active ingredients. This study was designed to test the assumption that the newly introduced insecticides kill mosquito vectors of malaria, and that their efficacy persists for at least 9 months. The design of this study and the methodology employed was based on the WHO protocol for the testing of insecticides [13]. A key reason for evaluating these insecticides was to determine the time taken to get to 100% post-exposure mortality, monthly for up to 12 months. The chemical manufacturers claimed delayed mortality up to five days. This work was conducted with the assistance of the KwaZulu-Natal Malaria Control Programme between 2021 and 2023.

## Methods

Syngenta Crop Protection AG provided the Actellic 300CS used in the study. Fludora®Fusion was provided by Bayer (Pty) Ltd and SumiShield 50WG was supplied by Philagro (Pty) Ltd. The positive control of DDT was supplied by the KwaZulu-Natal Provincial Department of Health.

### Study site

The study was conducted in the Ndumo (26° 54ʹ 43ʺ S 32° 15ʹ 48ʺ E) and Magwangwa (27° 23′ 13.6ʺ S 32° 04′ 37.3ʺ E) areas, located in northern KwaZulu-Natal. While these areas are well known for malaria, the houses selected had not been sprayed over the previous three years due to operational reasons. The members of the households still retained the knowledge of the principles and procedures of indoor residual spraying. They were aware that they should not paint, replaster, or wash the sprayed surfaces. Written consent was obtained from the head of the household to permit the spraying and for monthly evaluation of the sprayed surfaces.

### House selection

One hundred and sixty-eight (168) houses in the Ndumo and Magwangwa areas of KwaZulu-Natal were selected for the study. Ten houses of each of the four different surfaces, namely, galvanized steel with zinc (colloquially known as tin), mud, cement plastered, and painted surfaces, reflecting the building materials used in the area, were selected to be sprayed with the trial insecticides (10 trial huts × 4 surfaces × 3 insecticides = 120 huts). A further forty-eight (48) houses were selected to serve as the positive control (DDT sprayed) (6 per surface type) and the negative control (unsprayed surface) (6 per surface type).

### Bioassays for evaluating spray efficacy

Bioassays were carried out at monthly intervals following insecticide application. Cages of 3-day old, non-blood fed *An. arabiensis* females, sourced from well-established laboratory colonies were transported by road from the insectary at the South African Medical Research Council to the designated field site.

Standard WHO cones were applied to the walls of the test structures at 3 different heights (approximately 10 cm (bottom), 120 cm (middle) and 180 cm (top) above the floor) as recommended by WHO [13]. Thereafter 10 unfed female mosquitoes were introduced into each cone. Mosquitoes which were knocked down by the insecticide were counted at 30-min intervals for a period of an hour. At the end of the 30-min exposure period all 10 mosquitoes were returned to a holding cup and given access to a 10% sugar solution on cotton wool and revaluated at 60 min post exposure, to determine the knock-down effect of the insecticides. Thereafter, they were held in cups within an insectary (27 °C, 70% RH) for 24 h at which time the total number of survivors were determined. Observations continued every 24 h until 100% mortality was achieved.

### Insecticide application

The insecticides as supplied were mixed and applied according to their label instructions. Actellic 300CS used the application rate of 30 ml/m^2^ or 1 g ai/ m^2^. Fludora®Fusion used the application rate of 200 mg/m^2^ Clothianidin and 25 mg/m^2^ deltamethrin. SumiShield 50WG was sprayed at an application rate of 300 mg ai/m^2^. The positive control DDT 75% WP was applied using a rate of 2 g total DDT (1.44 g a.i.)/m^2^. All spray pumps used an 8002E with CFV nozzles. Application was done using calibrated vector control spray pumps, and a trained spray operator applied the insecticide to each structure. The spraying process was monitored by an expert spray supervisor.

### Data management

In the field, data collection was initially conducted using Microsoft Excel installed on a tablet. This approach allowed for easy entry and preliminary analysis of data on-site. Once the data collection phase was completed, the information was systematically transferred to REDCap [14], a more sophisticated and secure platform designed for managing longitudinal databases. This transfer to REDCap ensured enhanced data integrity and enabled complex data tracking over time. To ensure accuracy and reliability, data verification processes were rigorously applied. Each data entry made in REDCap was cross-checked against original records for inconsistencies or errors, a critical step in maintaining data quality.

Additionally, quality control measures were implemented both during the Excel phase and after the transfer to REDCap, including regular audits and validation checks to identify and correct any discrepancies. In REDCap advanced tools facilitated detailed analysis and reporting, enabling researchers to generate insightful and accurate reports, which were instrumental in interpreting the long-term trends and outcomes of the study.

### Statistical analysis

Mortality, defined as the proportion of mosquitoes dying, was observed over a period of 168 h. If mortality in the negative control exceeded 5% but was less than 20%, results were corrected using Abbott’s formula [15], however, this was not encountered.

The percentage mosquito mortality at each 24-h time point was averaged across the 12 months and described using the mean with standard deviation. Differences between treatments and the positive controls were tested using the Mann–Whitney statistic. Mixed effects logistic regression models were fitted to determine the fixed effects of treatment, substrate, and cone position on predicting whether 100% mortality is achieved at each 24-h time point. Where applicable, interaction effects between treatment and time, and between treatment, surface type and time, were included. Since data was clustered by house (three cones per house), random intercepts to account for the clustering of observations within houses were included. Odds ratios (ORs) with 95% confidence intervals are presented. The predicted probabilities of achieving 100% mortality were plotted. Data was analysed using Stata version 16 (StataCorp., College Station, TX, USA). Results were considered significant for p < 0.05.

## Results

At the end of the study period, the number of houses visited each month dropped from 168 to 151 since some participants opted out of the study, whilst in other cases the houses being used were destroyed, especially some mud structures which were destroyed by rain during the rainy season.

**Fig. 1:**
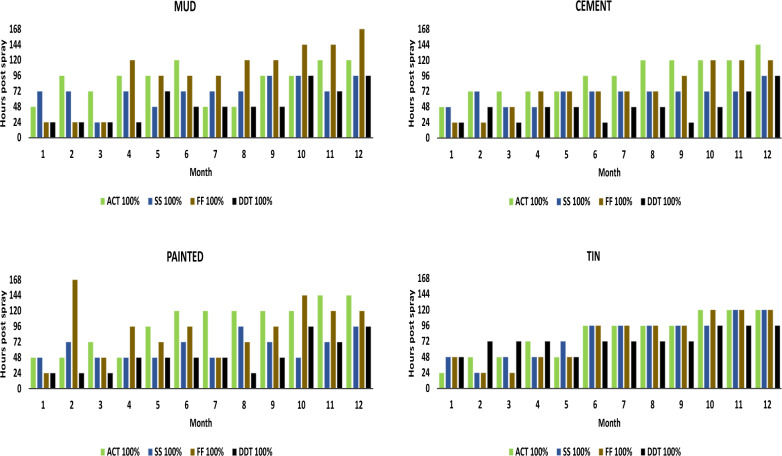
Number of hours required to achieve 100% mortality on the four types of sprayed surfaces

At month twelve, Actellic 300CS achieved an average knock-down rate of 29% on mud surfaces, 18% on cement surfaces and 19% on painted surfaces at the 60-min evaluation period. On the tin surface, Actellic 300CS averaged a knock down rate of 24%, Fludora®Fusion 17% and SumiShield 50WG 34% at the 60-min evaluation period. Regarding the activity achieved post-spray, the 12-month evaluation showed that it took Actellic 300CS 120 h to achieve 100% mortality across mud and tin surfaces and 144 h across paint and cement surfaces (Fig. 1). It took Fludora®Fusion up to 120 h and SumiShield 50WG up to 96 h to achieve 100% mortality on the tin surfaces.

The mean mosquito mortality rate at each time point is presented in Table 1. This table summarizes the activity at each observation period averaged over the 12-month period. Actellic showed significantly lower knockdown rates while Fludora®Fusion showed significantly higher knockdown rates when compared to the positive control, this was attributed to the insecticide combination in Fludora®Fusion. There was no significant difference in knockdown rates between SumiShield and the positive control, however, SumiShield reported significantly higher mortality rates for most time points when compared to the positive control.

**Table 1:** Mosquito knockdown and mortality (%) at each time point averaged over 12 months

	Timepoint (average of 12 months)								
	Knockdown [mean (standard deviation)]		Mortality [mean (standard deviation)]						
	30 min	60 min	24 h	48 h	72 h	96 h	120 h	144 h	168 h
Actellic	22.5 (27.5)	46.1 (33.2)	84.6 (19.9)	95.4 (9.3)	98.2 (6.7)	99.5 (5.3)	99.9 (1.9)	99.9 (1.3)	100 (0)
p-value^a^	< 0.001	< 0.001	0.034	0.1295	0.001	0.044	0.381	0.262	< 0.001
Fludora®Fusion	45.4 (31.2)	67.9 (28)	86.9 (18.2)	94.8 (10.5)	98.1 (5.9)	99.3 (4.3)	99.7 (3.3)	99.9 (2.9)	99.9 (2.9)
p-value^a^	< 0.001	< 0.001	0.925	0.01	< 0.001	< 0.001	0.333	0.195	0.111
SumiShield	28.3 (25.5)	52.2 30.4)	86.7 (19.6)	97 (8.2)	99.4 (3)	99.9 (1)	100 (0)	100 (0)	100 (0)
p-value^a^	0.154	0.144	0.612	0.001	0.015	0.26	0.001	0.001	0.001
Positive Control	31.2 (28.4)	54.4 (31.6)	86.3 (20.1)	95.5 (12.3)	98.3 (10.3)	99.1 (9.2)	99.2 (9.2)	99.2 (9.2)	99.2 (9.2)
Negative Control	0 (0.5)	0.1 (1.2)	0.9 (8.9)	1 (9)	1.1 (9.1)	1.2 (9.2)	1.3 (9.2)	1.4 (9.3)	1.7 (9.5)

^a^Mann–Whitney test comparing insecticide to the positive control

**Fig. 2:**
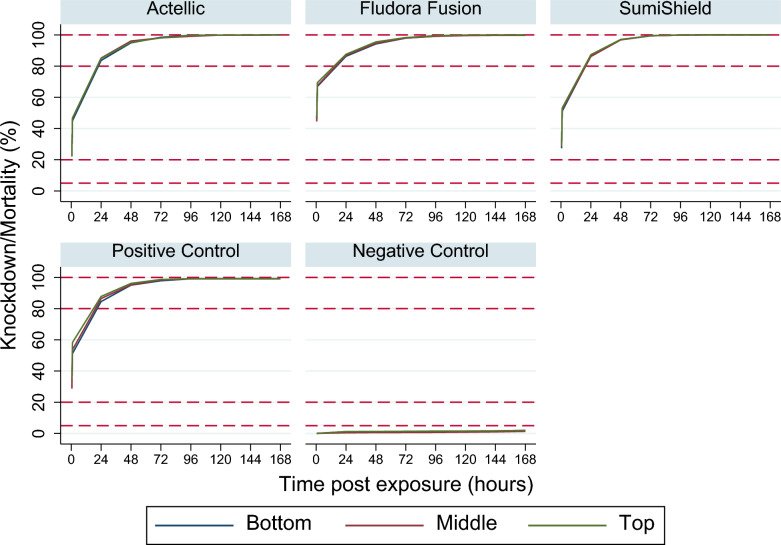
Mortality rates according to the height of the cone on the sprayed surface. The dashed lines indicate mortality rates at 5%, 20%, 80% and 100%

Figure 2 shows the knockdown and mortality rates by position. Across all insecticides and surface types, there were no apparent differences by position of cone on the sprayed surface. Therefore, the height of the cone above the floor did not influence mortality after exposure.

Table 2 presents the regression results comparing the efficacy of each insecticide against the positive control per surface type. No significant main effects and interaction effects were found for cone height and were not included in the models. Over time, SumiShield performed significantly better on mud (OR 2.28; 95% CI 1.72–3.04) and painted cement surfaces (OR 3.52; 95% CI 2.36–5.26) compared to the positive control. Actellic had lower odds of achieving 100% mortality on cement surfaces (OR 0.55; 95% CI 0.37–0.82) but higher odds on painted cement (OR 1.53; 95% CI 1.19–1.97) when compared to the positive control. There was no significant difference between Fludora®Fusion and the positive control except on tin surfaces (OR 0.67; 95% CI 0.47–0.95).

**Table 2:** Predictors of 100% mortality by surface type and the interaction between insecticide and time on the different sprayed surfaces

Predictor	OR (95% CI)			
	Mud	Cement	Painted cement	Tin
Insecticide				
Positive control	Reference	Reference	Reference	Reference
Actellic	0.69 (0.36–1.34)	2.12 (0.87–5.13)	**0.29 (0.14**–**0.6)**	0.97 (0.46–2.05)
Fludora®Fusion	0.73 (0.38–1.4)	1.77 (0.72–4.32)	0.76 (0.37–1.58)	**2.47 (1.17**–**5.21)**
SumiShield	**0.36 (0.18**–**0.73)**	1.47 (0.58–3.72)	**0.21 (0.09**–**0.49)**	2 (0.92–4.35)
Time (hours)	**3.25 (2.73**–**3.88)**	**6.92 (4.81**–**9.96)**	**2.97 (2.45**–**3.6)**	**6.5 (4.81**––**8.8)**
Insecticide*Time				
Actellic*Time	1.11 (0.9–1.38)	**0.55 (0.37**–**0.82)**	**1.53 (1.19**–**1.97)**	0.89 (0.63–1.27)
Fludora®Fusion *Time	0.88 (0.72–1.07)	0.67 (0.45–1.01)	1.03 (0.82–1.29)	**0.67 (0.47**–**0.95)**
SumiShield*Time	**2.28 (1.72**–**3.04)**	0.99 (0.63–1.57)	**3.52 (2.36**–**5.26)**	0.71 (0.49–1.02)
Month	**0.71 (0.69**–**0.73)**	**0.69 (0.67**–**0.71)**	**0.69 (0.67**–**0.71)**	**0.59 (0.56**–**0.61)**

OR (95% CI) are presented. Significant p-values (< 0.05) are indicated in bold

Table 3 portrays the logistic regression results comparing all insecticide and surface type combinations to the positive control applied to mud. Compared to the positive control on mud surfaces, SumiShield on all surfaces except tin had higher odds of 100% mortality. The positive control on cement also had significantly higher odds compared to the positive control on mud. Table 3 shows the significant interaction effects compared to the positive control on mud.

**Table 3:** Mixed effects logistic regression comparing all insecticides and surface types to DDT on mud

Predictors	Odds Ratio	95% CI	p-value
Insecticide and Surface			
Actellic Mud	0.69	0.26–1.87	0.469
Actellic Cement	1.4	0.59–3.36	0.445
Actellic Painted cement	0.77	0.34––1.71	0.516
Actellic Tin	0.79	0.36–1.73	0.562
Fludora®Fusion Mud	0.72	0.27–1.93	0.517
Fludora®Fusion Cement	1.2	0.47–3.08	0.706
Fludora®Fusion Painted Cement	2.1	0.86–5.12	0.105
Fludora®Fusion Tin	1.8	0.76–4.24	0.182
SumiShield Mud	**0.35**	**0.16**–**0.75**	**0.006**
SumiShield Cement	0.98	0.38–2.52	0.972
SumiShield Painted cement	0.57	0.21–1.51	0.256
SumiShield Tin	1.51	0.65–3.51	0.339
Positive Control Cement	0.66	0.16–2.72	0.565
Positive Control Painted cement	2.78	0.55–13.97	0.215
Positive Control Tin	0.82	0.31–2.18	0.693
Positive Control Mud	Reference		
Time (hours)	**3.44**	**2.11**–**5.61**	**< 0.001**
Insecticide and Surface*Time			
Actellic Mud	1.11	0.63–1.96	0.708
Actellic Cement	1.13	0.69–1.86	0.632
Actellic Painted cement	1.35	0.82–2.23	0.239
Actellic Tin	1.39	0.85–2.26	0.187
Fludora®Fusion Mud	0.87	0.5–1.52	0.627
Fludora®Fusion Cement	1.38	0.79–2.42	0.261
Fludora®Fusion Painted cement	0.9	0.54–1.49	0.678
Fludora®Fusion Tin	1.09	0.66–1.79	0.734
SumiShield Mud	**2.38**	**1.44**–**3.92**	**0.001**
SumiShield Cement	**2.05**	**1.15**–**3.63**	**0.014**
SumiShield Painted cement	**3.13**	**1.72**–**5.68**	**< 0.001**
SumiShield Tin	1.14	0.68–1.91	0.613
Positive Control Cement	**2.06**	**1.03**–**4.12**	**0.042**
Positive Control Painted cement	0.87	0.33–2.34	0.785
Positive Control Tin	1.54	0.89–2.67	0.12
Month	**0.68**	**0.65**–**0.7**	**< 0.001**

Significant ratios are in bold

Significant p-values (< 0.05) are indicated in bold

## Discussion

The evaluation was initiated to determine the efficacy of new generation insecticides for use in the malaria control programmes in southern Africa. Currently DDT and pyrethroids are used in the malaria control programme in South Africa. Actellic 300CS, Fludora®Fusion and SumiShield 50WG have all been recommended by the WHO for use in indoor residual spraying programmes [16], but have not been used in South Africa since there was no local data on residual efficacy. The insecticides were evaluated over a period of 12 months which is usually a single spray round in most African countries where the malaria transmission season is seasonal and runs from October to May. This study was conducted to determine the residual efficacy of Actellic 300CS, Fludora®Fusion and SumiShield 50WG on mud, cement, painted surfaces, and tin. The study has shown that all the insecticides tested can kill susceptible mosquito vectors of malaria in South Africa for up to nine months with increasing duration to achieve 100% mortality in subsequent months. Results also indicated that when applied to various substrates, Actellic 300CS was effective on all surfaces over a twelve-month evaluation period. Fludora®Fusion and SumiShield 50WG were effective over the twelve-month period when sprayed on all structure types. Nevertheless, over a span of 12 months, SumiShield showed the best efficacy and residuality on all structure types with a slight decrease on efficacy on tin structures. The results of the post-exposure mortality were encouraging when compared to DDT (the positive control) since delayed mortality was experienced when spraying these insecticides. All exposures to these insecticides usually resulted in 100% mortality within a 7-day period. A concern was raised that with delayed mortality occurring after 5 or 7 days, mosquitoes would still be able to lay eggs and take at least one more blood meal during the period that they survived. Chemical manufacturers claimed that exposure to insecticides induced feeding inhibition, but this is still to be demonstrated. Feeding inhibition after exposure to insecticides have been reported for other insects [17, 18].

The efficacy obtained in this study was corroborated by various other studies undertaken in many parts of Africa. In Kenya, the residual effect of Actellic 300CS lasted ten months on mud and concrete walls [19] whilst the current study showed mortality up to 12 months if the mosquitoes are evaluated beyond 24 h. Due to the long residual effect of pirimiphos-methyl, it is possible to achieve year-round protection with a single round of IRS. The results of the trial by [20] and [21] demonstrated that the residual efficacy of SumiShield™ 50WG extends up to nine months on all treated wall surface types, which would be suitable for countries where the main malaria transmission season lasts up to eight months. The long-lasting residual efficacy and unique mode of action of SumiShield™ 50WG suggests that it is an ideal product to be considered as a potential candidate insecticide formulation for IRS in malaria endemic countries. Indoor residual spraying with Fludora®Fusion induced high and prolonged mortality of malaria vectors for 7–10 months [22]. However, in a study by Fongnikin et al*.* [23] Fludora®Fusion showed delayed mortality rates above the WHO’s 80% threshold over a period of 11 months. The study in South Africa demonstrated that Fludora®Fusion is an important addition to the current portfolio of IRS.

The present study highlights the need for assessing mosquito mortality beyond the currently recommended 24 h post exposure. Failure to do so may lead to underestimation of the residual efficacy of IRS products, as delayed mortality will lead to a further reduction in mosquito vector populations and potentially negatively impact disease transmission. Determination of residual activity of insecticides is essential information for the selection of appropriate indoor spraying operations. Current data suggest variable durations of spray cycles for each product, according to the type of wall surfaces, highlighting the importance of testing candidate products in local contexts before using them on a large scale. A study in South Cameroon found that bendiocarb is very effective on cement and wood whilst lambda-cyhalothrin was effective on wood [24]. This same study found that Deltamethrin had a good residual life on cement. However, due to the high levels of resistance to pyrethroids found across the continent the use of a pyrethroid in combination with a new active ingredient does raise some concerns regarding the development of resistance to the combination insecticide.

Variations in the effectiveness of insecticides in the field are not uncommon and results of a short-term study cannot define effectiveness of the insecticides that are being evaluated. The current study found that Actellic 300CS, Fludora®Fusion and SumiShield 50WG had a good residual life on tin structures. SumiShield exhibited a good residual life when tested on cement, mud, and painted surfaces.

The evaluation achieved its objective in determining the residual life of the insecticides in houses of the community living in the Ndumo and Magwangwa area using the WHO cone bioassay method. The results showed that the trial insecticides met the WHO standards of > 80% mortality for at least 12 months and has comparable residual efficacy in relation to the currently used insecticide (DDT) by the malaria control programme. Therefore, Actellic 300CS, Fludora® Fusion and SumiShield 50WG can form one of the tools in an integrated vector control programme in South Africa since it has equivalent effectiveness to DDT and is environmentally acceptable.

All the new generation insecticides evaluated are suitable for indoor residual spraying. SumiShield demonstrates versatile application potential, adhering effectively to various surfaces and exhibiting high efficacy. This is the insecticide to use if all structures need to be sprayed with a single insecticide. Actellic, while effective, shows it is most effective on painted cement surfaces whilst Fludora®Fusion is best on tin surfaces. These insecticides offer flexibility in usage, either through rotational application or in a mosaic pattern. To mitigate the swift emergence of insecticide resistance, it is imperative to establish and implement an insecticide resistance management plan before these insecticides are put into use.

## Conclusion

All the insecticides evaluated are suitable for use in an indoor residual spray programme as they can induce delayed mortality for up to twelve months on most surfaces. To varying degrees, these insecticides are effective on all the sprayed surfaces tested. These insecticides can be used in an insecticide resistance management strategy either in rotation or in mosaic spraying. SumiShield was found to be most effective on all surfaces except for tin surfaces. All three insecticides are suitable replacements for DDT in indoor residual spray programmes.

## Data Availability

The datasets used and/or analysed during the current study are available from the corresponding author on reasonable request.

## Abbreviations

CS: Capsule suspension
DDT: Dichloro-diphenyl-trichloroethane
WHO: World Health Organization
IRS: Indoor residual spraying
OR: Odds ratio
REDCap: Research electronic data capture
WG: Wettable granule
